# Angiogenesis in Adipose Tissue: The Interplay Between Adipose and Endothelial Cells

**DOI:** 10.3389/fphys.2020.624903

**Published:** 2021-02-09

**Authors:** Jacqueline Herold, Joanna Kalucka

**Affiliations:** ^1^Department of Biomedicine, Aarhus University, Aarhus, Denmark; ^2^Aarhus Institute of Advanced Studies (AIAS), Aarhus University, Aarhus, Denmark

**Keywords:** adipocytes, adipose tissue, angiogenesis, endothelial cells, obesity

## Abstract

Obesity is a worldwide health problem, and as its prevalence increases, so does the burden of obesity-associated co-morbidities like type 2 diabetes or cardiovascular diseases (CVDs). Adipose tissue (AT) is an endocrine organ embedded in a dense vascular network. AT regulates the production of hormones, angiogenic factors, and cytokines. During the development of obesity, AT expands through the increase in fat cell size (hypertrophy) and/or fat cell number (hyperplasia). The plasticity and expansion of AT is related to its angiogenic capacities. Angiogenesis is a tightly orchestrated process, which involves endothelial cell (EC) proliferation, migration, invasion, and new tube formation. The expansion of AT is accelerated by hypoxia, inflammation, and structural remodeling of blood vessels. The paracrine signaling regulates the functional link between ECs and adipocytes. Adipocytes can secrete both pro-angiogenic molecules, e.g., tumor necrosis factor-alpha (TNF-α), interleukin-6 (IL-6), or vascular endothelial growth factor (VEGF), and anti-angiogenic factors, e.g., serpins. If the pro-angiogenic molecules dominate, the angiogenesis is dysregulated and the endothelium becomes dysfunctional. However, if anti-angiogenic molecules are overexpressed relative to the angiogenic regulators, the angiogenesis is repressed, and AT becomes hypoxic. Furthermore, in the presence of chronic nutritional excess, endothelium loses its primary function and contributes to the inflammation and fibrosis of AT, which increases the risk for CVDs. This review discusses the current understanding of ECs function in AT, the cross-talk between adipose and ECs, and how obesity can lead to its dysfunction. Understanding the interplay of angiogenesis with AT can be an approach to therapy obesity and obesity-related diseases such as CVDs.

## Introduction

Recent findings have led us to reconsider the notion of adipose tissue (AT) being a mere storage depot for body energy. Instead, ATs are emerging as endocrine and immunologically active organs with multiple effects on the regulation of systemic energy homeostasis (Lee et al., 2013). AT is classified as either white (WAT), brown (BAT), or beige/brite based on whether it functions as energy storage or thermogenic organ. Adipocytes in BAT are rich in mitochondria and generate chemical energy in the form of heat (Sun et al., 2020). BAT expands when an organism is exposed to prolonged cold conditions and is linked with increased insulin sensitivity (Cypess et al., 2009; Van Marken Lichtenbelt et al., 2009). WAT stores energy in the form of triglycerides in lipid droplets and is an endocrine organ that releases hormones, growth factors, and adipokines such as leptin and adiponectin. AT consists of not only adipocytes (40–50%) but also connective tissue matrix, vascular and neural cells, and non-adipocyte cells called stromal vascular fraction (SVF). SVF includes preadipocytes, immune cells (macrophages, natural killer cells, B-lymphocytes, and T-lymphocytes), endothelial cells (ECs), vascular progenitors, fibroblasts, and mesenchymal stem cells (Rosenwald and Wolfrum, 2014). The WAT is mainly divided into two types based on its distribution site, the subcutaneous (SAT) and visceral AT (VAT). During overnutrition, the surplus energy is stored as triglycerides in the SAT leading to AT expansion. AT generates new adipocytes (hyperplasia) and enlarges existing adipocytes (hypertrophy; Hafidi et al., 2019). These processes change the structure and function of AT, and these events are defined as “AT remodeling” (Longo et al., 2019).

The prevalence of obesity has increased all over the world during the last 50 years and more than 650 million obese adults were reported in 2016. According to the World Health Organization (WHO), obesity is an “abnormal or excessive fat accumulation that may impair health.” The general measurement of obesity is the body mass index (BMI). Subjects with a BMI ≥ 25 kg/m^2^ are classified to be overweight, while those with a BMI ≥ 30 kg/m^2^ are considered obese. A major cause of obesity is the imbalance between energy intake and expenditure, resulting in the storage of triglycerides in AT. Many factors, including genetics, epigenetics, ethnicity, and environmental factors are involved in the complex pathogenesis of obesity (Rohde et al., 2019). Obesity is a critical risk factor for many diseases, including type 2 diabetes, non-alcoholic fatty liver disease, hypertension, and cardiovascular disease (CVD); it is also linked to several cancers (Ungefroren et al., 2015). In obesity, alteration in AT remodeling may induce the dysregulation of AT-secreted adipokines and cytokines, and the increased secretion of pro-inflammatory molecules may promote systemic low-grade inflammations (Mancuso, 2016). This results in macrophage and T-cell infiltration into AT (Huh et al., 2014). However, AT remodeling does not necessarily lead to these obesity-related diseases; several studies have reported that a subgroup of obese individuals are metabolically “healthy” and display normal physiology and biomarker profiles (Blüher, 2010). This suggests the presence of differences in pathways that regulate AT dysfunction, which further leads to dysfunction in other organs.

The vascular circulatory system forms an extensive network of arteries, veins, and capillaries and is important for continuous supply and delivery of nutrients and oxygen to all tissues (Aird, 2004). The blood vessels are lined with a thin layer of ECs, the main drivers of sprouting angiogenesis (Eelen et al., 2018). In healthy adult tissue, ECs remain quiescent. The switch from quiescent to proliferative/angiogenic ECs is mediated by changes in metabolism and angiogenic factors (Draoui et al., 2017; Kalucka et al., 2018). Angiogenesis requires a balance between pro-angiogenic and anti-angiogenic molecules. Overexpression of pro-angiogenic molecules can also lead to EC dysfunction. Dysfunctional ECs cannot induce normal angiogenesis, which leads to lesser blood vessel formation. Lower vascular density is a high-risk factor for hypoxia and inflammation and is associated with various diseases, such as CVDs, obesity, and cancer (Eelen et al., 2018; Nijhawans et al., 2020).

**Figure 1:**
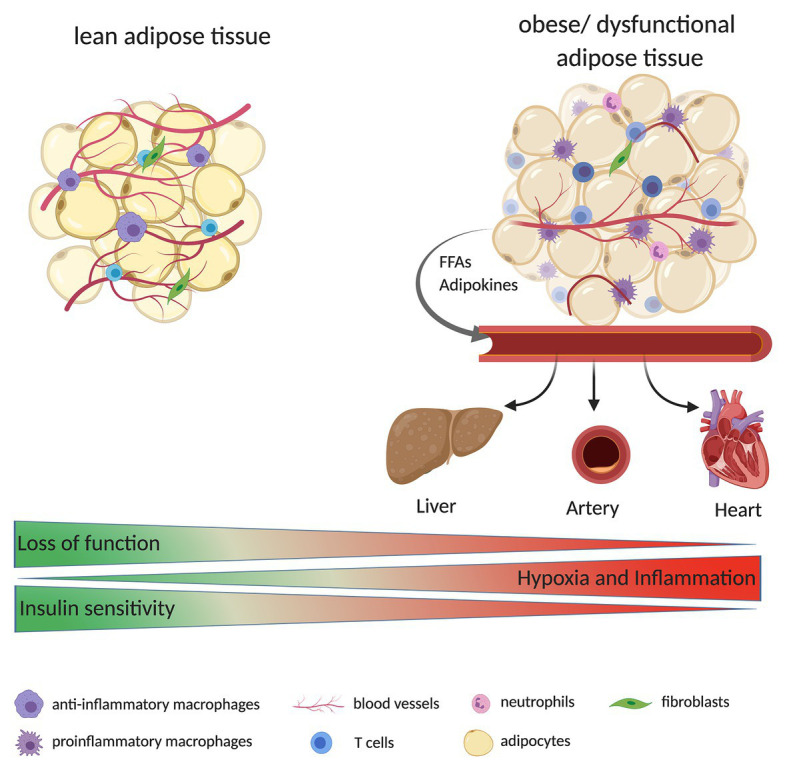
Adipose tissue (AT) is composed of multiple cells, including adipocytes, immune cells (e.g., macrophages, T cells, and neutrophils), fibroblasts, and endothelial cells (ECs; building blocks of blood vessels). During the development of obesity, AT expands quickly. Due to decreased tissue vascularization, increased hypoxia, and inflammation, AT might become dysfunctional. Dysfunctional AT releases high levels of free fatty acids (FFAs) and adipokines (e.g., leptin) to the bloodstream, reaching other organs such as liver, heart, and large arteries. Ultimately this can result in a higher fat accumulation in these tissues and alter their function e.g., the organs are less insulin sensitive or display an increase in immune cell infiltration/inflammation.

Obesity is accompanied by EC dysfunction and decreased vascular density (Figure 1). This is mainly due to disbalance and overexpression of pro-angiogenic and pro-inflammatory stimulus. Therefore, understanding the precise relationship between adipocytes and ECs are of significant importance. In this review, we have discussed the function of ECs in AT with respect to the influence of obesity on angiogenesis, the crosstalk between adipocytes and ECs, and how obesity can influence ECs (dys)function.

## Angiogenesis in Healthy Adipose Tissue

Adipose tissue is a highly vascularized tissue, and the density of blood vessels is important for the regulation of adipocyte function and adipogenesis. In addition to nutrients and oxygen, the vessels also transport growth factors, cytokines, and hormones that are required for adipocyte function, growth, and survival (Cao, 2013). Furthermore, the vessels regulate the transport of adipokines, cytokines, and growth factors from the AT to other organs and thus promote the endocrine function of the AT. The vascular system controls alteration in the AT microenvironment, including acidosis and hypoxia, which influences the adipocyte function, preadipocyte differentiation, and the AT mass (Cao, 2013; Figure 1). There are different possible triggers for angiogenesis in AT, the angiogenic expansion may be due to signals emanating from proliferating and enlarging adipocytes or it may be triggered by metabolic signals with the enlargement of adipocytes as a parallel phenomenon (Corvera and Gealekman, 2014). These processes are not mutually exclusive, and a combination of the two is most probably responsible for angiogenesis. The expansion of AT is angiogenesis-dependent (Rupnick et al., 2002). With a high nutrient availability, the adipocytes store lipids in lipid droplets, and as their size expands oxygen availability is reduced. This mild hypoxic status can induce angiogenesis and remodeling of extracellular matrix to reduce hypoxia (Crewe et al., 2017). In healthy AT, the vessels are lined by a single monolayer of quiescent ECs, which can rapidly switch to the angiogenic/proliferative state in the presence of angiogenic and metabolic signals to form new blood vessels (Draoui et al., 2017). AT produces and secretes various angiogenic factors such as angiopoietin-2 (Angpt2) and vascular endothelial growth factor (VEGF) as well as adipokines such as leptin and adiponectin, which influence and modulate angiogenesis and the vascular structure (Cao, 2010; Sorop et al., 2017). This suggests an autoregulatory function for angiogenesis in AT (Cao, 2007).

Interestingly, studies show the presence of precursor cells in the vessel wall, which have the capacity to differentiate into ECs and/or adipocytes in WAT and BAT depots (Tran et al., 2012). Tang et al. identified the presence of white adipocyte progenitors in the mural compartment of adipose vasculature, but not in the vasculature of other tissues. These mural cells had high adipogenic potential (Tang et al., 2008). These results indicate a link between ECs and adipocytes in terms of their interchangeability in the presence of a possible “switch” and the cell–cell interaction.

## Angiogenesis in Obesity

Obesity is characterized by a rapid expansion of AT, which affects tissue vascularization. The lack of vessels leads to decreased oxygen supply in adipocytes, which leads to hypoxia that promotes inflammation (Hodson et al., 2013), and inadequate vessel maintenance and growth. During hypoxia, the hypoxia-inducible factors (HIFs) signaling is activated (Wood et al., 2009). Once translocated to the nucleus, HIF1α dimerizes with HIF1β and forms the functional transcription factor HIF1. HIF1 can bind to hypoxia response elements of target genes, including VEGFA and Angpt2, which induce angiogenic response (Tahergorabi and Khazaei, 2013). HIF1α is reportedly increased in the AT of obese patients and its expression was found to be reduced after surgery-induced weight loss (Lemoine et al., 2013). Genetic deletion of HIF1α in adipocytes decreases the risk of obesity-induced inflammation and insulin resistance (Lee et al., 2014). Hypoxia stimulates inflammation and results in the accumulation of macrophages and other immune cells (Murdoch et al., 2005). Activation of HIF-signaling pathways in macrophages in obese mice leads to induction of platelet-derived growth factor (PDGF) expression, which is likely to induce the tube formation of ECs to improve vascular density (Pang et al., 2008). During inflammation, several factors released from immune cells, such as tumor necrosis factor alpha (TNFα; Madge and Pober, 2001), act on ECs activating the signaling pathway of factor nuclear kappa B (NF*κ*B; Benelli et al., 2007). Additionally, with an increase in angiogenesis, more blood vessels are formed to provide oxygen and nutrients for the metabolic needs of the cells present at the inflammatory sites (Costa et al., 2007).

During obesity, there is a higher release in free fatty acids (FFAs) from adipocytes into the bloodstream, owing to a saturated storage capacity and dysfunctional adipocyte metabolism (Hafidi et al., 2019; Figure 1). The FFAs can be taken up by ECs through either fatty acid transport proteins 3 and 4 (FATP3/FATP4) or the scavenger receptor cluster of differentiation CD36 (CD36) and can be converted into hydrophilic acyl-CoAs. These are involved in several processes, including the synthesis of ceramides, which are membrane components. Furthermore, FFAs can activate the NF-κB through Toll-like receptor (TLR) signaling (Goldberg and Bornfeldt, 2013), which in turn can activate the process of inflammation (Theodorou and Boon, 2018).

The VEGF/VEGF-receptor (VEGFR) system is the main regulator of the angiogenic activity in AT and is expressed in the SVF and mature adipocytes (Lemoine et al., 2013). In a murine model with a *VEGF* deletion in AT, the reduced vascular density accompanied by enhanced hypoxia, inflammation, and apoptosis was observed (Sung et al., 2013). VEGF binds to its tyrosine kinase receptors VEGFR1 and VEGFR2 in order to perform various biological functions. VEGFA plays the role in AT angiogenesis and its expression is increased during the adipocyte differentiation (Fukumura et al., 2003). VEGF secreted by AT also stimulates the proliferation of vascular smooth muscle cells (VSMCs). Schlich et al. (2013) demonstrated that VSMC cultivated in the adipocyte-conditioned medium had a higher VEGFR1 and VEGFR2 expression, as well as higher VEGF secretion. Although VEGF binds to both receptors, VEGFR2 mediates most of the cellular responses to induce migration, survival, and proliferation of ECs (Costa et al., 2007). Blocking VEGFR2 limits diet-induced AT expansion by decreasing angiogenesis and adipogenesis (Tam et al., 2009). However, VEGFR2 has an opposite effect in lymphatic vessels (Zhang et al., 2018). Mice with a genetic deletion of neuropilin 1 (Nrp1) and Vegfr1 are resistant to diet-induced obesity by reduced lacteal chylomicron uptake. The inhibition of Vegfr2 restores the permeable junction and rescued chylomicron transport and the mice are not resistant to diet-induced obesity anymore (Zhang et al., 2018).

Overexpression of VEGF in WAT and BAT in mice led to increased number and size of blood vessels, increased insulin sensitivity, and improved glucose tolerance (Elias et al., 2012). The transgenic mice were protected from diet-induced obesity and local hypoxia, which was indicated by decreased expression of HIF1. Lu and Zheng (2013) reported that VEGF repression in mice also leads to resistance of diet-induced obesity and surprisingly higher expression of BAT markers, including uncoupling protein 1 (UCP1) and Cell Death Inducing DFFA Like Effector A (CIDEA; Lu et al., 2012). VEGFB was found to be expressed in ECs of the skeletal muscles, heart, and BAT. It binds to VEGFR1 and Nrp1 and increases the expression of FATP3/FATP4 to induce the FFA uptake (Hagberg et al., 2010). Another study demonstrated that the binding of VEGFB to VEGFR1 results in the activation of VEGF/VEGFR2 pathway and improves the capillary density and insulin signaling in AT (Robciuc et al., 2016).

Genetic deletion, as well as pharmacological inhibition of endothelial VEGFR1, increased adipose angiogenesis and browning of SAT, leading to elevated thermogenesis (Seki et al., 2018). Additionally, the anti-VEGFR1 treatment led to higher expression levels of UCP1 and smaller adipocyte size in WAT (Seki et al., 2018). This implied the beginning of browning in WAT, which effected the energy expenditure. A high-fat diet in transgenic mice with a deletion of VEGFR1 in ECs resulted in reduced body weight and body fat mass. Furthermore, the VEGFR1 knockout significantly ameliorated obesity-induced dysfunction by lowering the levels of FFAs, glycerol, triglyceride, glucose, and insulin in the blood of these mice (Seki et al., 2018). These results demonstrate the regulatory role and crosstalk between the different VEGF-members and their receptors that are involved in various mechanisms (Elias et al., 2013). Also, the potential of anti-angiogenic compounds for the treatment of obesity was investigated. Angiostatin and endostatin, two inhibitors of angiogenesis, inhibit weight gain in ob/ob mice and diet-induced obesity (Rupnick et al., 2002). Endostatin inhibits dietary-induced obesity and adipogenesis *via* decreased expression of mTOR. Furthermore, treatment with endostatin had a preventive effect on obesity-induced complication such as glucose intolerance (Hui et al., 2015).

In addition to VEGF, other angiogenic factors are expressed in AT. For example, PDGF is expressed in all cell types of AT (preadipocytes, adipocytes, macrophages, and ECs), however, its expression levels may differ (Engin, 2017). Preadipocytes express more PDGF than mature adipocytes. However, since in obesity, most of the preadipocytes are differentiated into mature adipocytes, the number of preadipocytes is reduced and thus the local PDGF level decreases. To meet the demand for PDGF, AT macrophages increase the PDGF production. In obese subject, in response to the reduced vascular density in AT and increased hypoxia, macrophages express PDGF to facilitate capillary formation. It is mandatory that the balance between these two angiogenic factors is maintained in order to form functional new capillaries. Angiogenesis is coordinated by VEGF and PDGF through their related receptors on ECs and VSMCs, respectively (Greenberg et al., 2008). The expression of another angiogenic factor, fibroblast growth factor-2 (FGF-2), is increased during adipocyte differentiation and during the induction of obesity by high-fat diet in mice. In AT, FGF2 enhanced the inflammation response through NLRP3 inflammasome activation (ZhuGe et al., 2020).

Peroxisome proliferator-activated receptor gamma (PPARγ), the master regulator of adipocyte differentiation, can influence the angiogenesis. Multiple studies demonstrate that PPARγ inhibits proliferation of ECs, however, in AT angiogenesis was enhanced by PPARγ activators (Gealekman et al., 2008). AT obtained from mice and humans treated with PPARγ agonist *rosiglitazone* were found to exhibit increased capillary density and capillary sprouting (Gealekman et al., 2012). Co-culture experiments of adipocytes and ECs demonstrated that the PPARγ expression levels were lower in EC–adipocyte coculture than that in the adipocyte-only control, and smaller lipid droplets per adipocyte were found in the latter (Hammel and Bellas, 2020). Other studies described anti-inflammatory effects of PPARγ in ECs including inhibition of NF-*κ*B signaling, decreased expression of chemokines, and proinflammatory adhesion molecules, including intercellular adhesion molecule (ICAM)-1 and vascular cell adhesion molecule (VCAM)-1 (Mehrotra et al., 2014). Mice with a knockdown of PPARγ in ECs that were fed a high-fat diet were found to have decreased SAT and VAT mass but increased spleen and liver weights compared to those in control mice, in spite of same body weight in both groups. Moreover, the adipocyte size was 25% lower in VAT from PPARγ knockdown mice compared to control mice (Kanda et al., 2009). Furthermore, the knockdown mice had lower glucose and insulin levels and were more insulin sensitive. However, these mice showed a higher concentration of circulating FFA, increased hepatic CD36 expression, and increased very low-density lipoprotein (vLDL) production. Taken together, these studies demonstrate that PPARγ in ECs contributes to metabolic response in various organs.

## Metabolic Disorders Related to Obesity and Cardiovascular Complications

With the development of obesity, AT becomes dysfunctional. In obesity, a higher release of FFAs from the adipocytes into the bloodstream is observed. FFAs from the VAT can drain directly to the liver *via* the portal circulation, where they affect the hepatocytes. The FFAs reduce insulin degradation, resulting in hyperinsulinemia, and induce insulin resistance thereby increasing glucose production in the liver (Lee et al., 2013). Furthermore, the higher amount of circulating FFAs provides more substrate for triglyceride synthesis leading to a higher production of vLDL, which is atherogenic (Cepeda-Valery et al., 2011), thus contributing to hyperlipidemia, hepatic steatosis, and nonalcoholic fatty liver diseases (Hijona et al., 2010). The higher production of vLDL and low-density lipoproteins (LDL) results in a higher release of these molecules to the circulation, which in turn leads to their retention in the intima within the artery wall; further, the oxidation of LDL contributes to EC activation (Hansson, 2005). The adhesion of leukocytes in the artery wall and a superficial erosion of ECs, leads to the formation of a platelet thrombus. The microvessels become more and more fragile and are potential locations for hemorrhage and thrombosis (Costa et al., 2007). This eventually leads to atherosclerosis. As previously mentioned, absorbed FFAs in ECs are converted to acyl-CoAs that are involved in the synthesis of ceramides and sphingolipids (De Lima et al., 2019). These lipid classes were reported to be positively correlated with cardiovascular disease and cardiovascular-related death in a subcohort of the Long-Term Intervention with Pravastatin in Ischemic Disease (LIPID) study, which included individuals with a myocardial infarction or hospital admission for unstable angina, for a randomized trial of *pravastatin* (Mundra et al., 2018). Fatty acid uptake by the ECs involves crosstalk between the ECs and AT. Bae et al. (2020) showed that Angpt2, which is highly expressed in SAT, can regulate the fatty acid uptake in ECs; ECs treated with Angpt2 showed increased fatty acid uptake *via* the scavenger receptor CD36 and FATP3 in SAT. Angpt2 activates the integrin α5β1 signaling pathway, which results in a translocation of CD36 and FATP3, thereby improving the fatty acid uptake in SAT. Moreover, Angpt2 overexpression in AT results in an increased expression of angiogenic and endothelial markers, VEGFA and CD31, and an increased vascular density in SAT (An et al., 2017). Furthermore, under a high-fat diet, mice with endothelial-specific knockout of Angpt2 exhibited in improved glucose clearance and insulin sensitivity, as shown by oral glucose tolerance test (oGTT) and insulin tolerance test (ITT). The Notch signaling *via* Rbp-j*κ* activation plays a role in fatty acid transport in the heart. The inhibition of Notch signaling results in decreased expression of endothelial lipase, CD36, and FATP4 (Jabs et al., 2018). This alteration leads to a switch from fatty acids to glucose as sources for energy production, which is known from several animal models of heart failure.

Obesity is also associated with diabetes, and the expression of p53 in ECs was found to be increased in the diabetic state. Under the high-calorie diet, the EC-specific p53 knockout mice showed improved insulin sensitivity and glucose tolerance along with lower plasma insulin levels as well as VAT and SAT (Yokoyama et al., 2014). The deletion of p53 results in higher oxygen consumption and higher glucose uptake in skeletal muscles and BAT through the upregulated endothelial expression of glucose transporter (Glut)1. Moreover, the deletion of p53 resulted in higher expression of genes related to mitochondrial biogenesis, such as PPARγ coactivator-1α (PGC1α), *via* higher phosphorylation of endothelial nitric oxide synthase (eNOS) in skeletal muscles. Conversely, the upregulation of endothelial p53 caused metabolic abnormalities. PGC1α has widespread functions in different tissues and cell types (Sawada et al., 2014), and diabetes induces the expression of PGC1α in ECs (Carmeliet and Jain, 2011). Endothelial-specific PGC1α-overexpression resulted in significantly repressed EC migration, a hallmark of EC function, as measured by transwell migration assays. This inhibition of EC migration is induced by the activation of Notch-signaling and inhibition of Rac/Akt/eNOS signaling, suggesting that PGC1α mediates, in part, the vascular dysfunction caused by diabetes.

Under the high-fat diet, the insulin signaling in ECs is impaired, showing a reduction in insulin receptor substrate-2 (Irs2) and insulin-induced eNOS phosphorylation. This leads to a further reduction in insulin-induced glucose uptake by the skeletal muscles triggered by decreased capillary recruitment and interstitial insulin concentration in the skeletal muscles (Kubota et al., 2011). The reduction of Irs2 in ECs also influences the insulin secretion in the pancreatic β-cells by impairing islet blood flow (Hashimoto et al., 2015). The insulin increase NO synthesis, *via* Phosphoinositide 3-kinase (PI3-K), in ECs, and platelets and in VSMCs. Mediated by nitric oxide, insulin increases the protein expression and secretion of VEGF (Doronzo et al., 2004). Hasan et al. (2020) demonstrated that obesity leads to higher activation of Notch signaling in ECs. This results in decreased insulin sensitivity and lower glucose uptake in the muscles. These effects are mediated by inhibition of genes involved in caveolae formation, such as caveolar protein caveolin 1 (Cav1), and results in a diminished insulin transport to the muscles.

**Figure 2:**
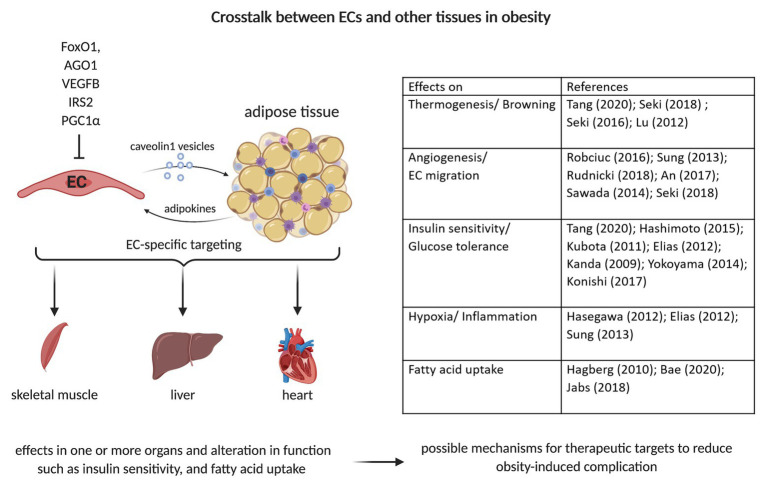
Crosstalk between ECs and adipocytes/adipose tissue. Caveolin 1 (Cav1)-vesicle can transport small molecules from ECs to adipocytes. The EC-specific depletion/deletion of various proteins such as Forkhead Box O1 (FoxO1), Argonaute (AGO1), vascular endothelial growth factor (VEGF), Insulin Receptor Substrate 2 (IRS2), or PPARG Coactivator 1 Alpha (PGC1α) can alter adipocytes’ function. This can result in changes of fatty acid uptake, insulin signaling, and glucose homeostasis. This can further induce alterations in other organs’ functions, such as liver, heart, and skeletal muscle. The crosstalk with other organs is a possible therapeutic target mechanism to reduce obesity-induced complications, e.g., insulin resistance and fat accumulation in other organs.

Extracellular vesicles (EVs) can be involved in cell-to-cell and organ-to-organ communication *via* metabolic signals. This has been demonstrated for e.g., adipose-derived stem cells EVs by Zhao et al. (2018). Obese mice treated with these exosomes showed improved insulin sensitivity. EVs of AT macrophages from lean mice injected to obese mice caused whole-body insulin sensitivity and glucose tolerance, as determined by GTT and ITT (Ying et al., 2017). Conversely, EVs from obese mice injected to lean mice led to insulin resistance, lower glucose tolerance, and increased inflammation. This outcome was induced by miRNA155, which targets PPARγ and impairs the expression of Glut4. Crewe et al. (2018) demonstrated crosstalk between ECs and adipocytes, *via* Cav1-containing adipocyte EVs that are taken up by ECs (Figure 2). This transfer is dependent on metabolic status and increases during fasting. Proteomic analysis of the EVs showed an increase in the expression of proteins involved in polyamine metabolism, antioxidant response, and transport of small molecules during the fasted state. However, a decrease in molecules involved in lipid and amino acid metabolism was observed (Crewe et al., 2018). There is further evidence of direct crosstalk between ECs and adipocytes/AT. The endothelial-specific knockdown of the transcription factor Forkhead Box O1 (FoxO1) led to a higher vascular density in AT (Rudnicki et al., 2018). Under the high-fat diet, VAT displays a higher expression of Pecam1, higher capillary number per adipocyte, reduced triglycerides in blood and less hepatic lipid accumulation. FoxO1 depletion increases the ECs proliferation by upregulating glycolytic markers, including Glut1, hexokinase 2, and phosphofructokinase. The suppression of argonaute (AGO1), a regulator of the endothelial hypoxia response, leads to a desuppression of VEGFA and thereby contributing to hypoxia-induced angiogenesis (Chen et al., 2013). The EC-specific AGO1 knockout in mice resulted in lower body weight gain under a high-fat high-sucrose diet, which could be explained by the lower SAT and BAT mass (Tang et al., 2020; Figure 2). The knockout mice had lower fasting glucose level and higher insulin sensitivity, as confirmed by oGTT, ITT, and higher phosphorylation levels of AKT and AMPK, hallmarks of insulin sensitivity, in SAT and BAT. Furthermore, browning in SAT was observed, as indicated by increased expression levels of UCP1 and its regulator PGC1α. Moreover, AGO1 deletion led to increased levels of VEGFA and higher CD31+ staining in SAT (Tang et al., 2020).

## Conclusion

Obesity can lead to many complications and comorbidities. It affects not only the AT but also several organs such as the liver or cardiovascular system. EC dysfunction is a hallmark of obesity. For a long time, it was thought that only the AT affects the EC function. However, several studies have demonstrated that changes in EC functions and signaling pathways influence adipocyte metabolism and can improve the obesity-induced dysfunction (Figure 2). These first insights and differences in the metabolism of healthy and dysfunctional ECs could provide a basis for further studies that may target ECs for therapeutic benefits. The EC-specific treatment can potentially have direct effects on the metabolism of adipocytes and/or AT. However, further studies are necessary to understand the direct crosstalk between ECs and adipocytes as well as the organ-to-organ communication that may help develop strategies to reduce the obesity-induced complications and alteration to whole body metabolism.

## Author Contributions

JH and JK selected the literature, wrote the manuscripts, and prepared the figures. All authors contributed to the article and approved the submitted version.

### Conflict of Interest

The authors declare that the research was conducted in the absence of any commercial or financial relationships that could be construed as a potential conflict of interest.
